# Perspectives on Bulk-Tissue RNA Sequencing and Single-Cell RNA Sequencing for Cardiac Transcriptomics

**DOI:** 10.3389/fmmed.2022.839338

**Published:** 2022-02-22

**Authors:** Jana-Charlotte Hegenbarth, Giuliana Lezzoche, Leon J. De Windt, Monika Stoll

**Affiliations:** ^1^ Department of Molecular Genetics, Faculty of Science and Engineering, Faculty of Health, Medicine and Life Sciences, Maastricht University, Maastricht, Netherlands; ^2^ Department of Biochemistry, CARIM School for Cardiovascular Diseases, Maastricht University, Maastricht, Netherlands; ^3^ Department of Genetic Epidemiology, Institute of Human Genetics, University Hospital Münster, Münster, Germany

**Keywords:** sequencing, single-cell, bulk-seq, transcriptomics, heart

## Abstract

The heart has been the center of numerous transcriptomic studies in the past decade. Even though our knowledge of the key organ in our cardiovascular system has significantly increased over the last years, it is still not fully understood yet. In recent years, extensive efforts were made to understand the genetic and transcriptomic contribution to cardiac function and failure in more detail. The advent of Next Generation Sequencing (NGS) technologies has brought many discoveries but it is unable to comprehend the finely orchestrated interactions between and within the various cell types of the heart. With the emergence of single-cell sequencing more than 10 years ago, researchers gained a valuable new tool to enable the exploration of new subpopulations of cells, cell-cell interactions, and integration of multi-omic approaches at a single-cell resolution. Despite this innovation, it is essential to make an informed choice regarding the appropriate technique for transcriptomic studies, especially when working with myocardial tissue. Here, we provide a primer for researchers interested in transcriptomics using NGS technologies.

## Introduction

The heart is the first fully functional organ to be formed during embryonic development. In the past decades, conventional molecular biology studies have revealed important physiological and pathological mechanisms within the cardiovascular system, however it was unable to comprehend the finely orchestrated interactions between the various cells and cell types within the heart. Thus, our understanding of cardiac cell diversity remains limited and cardiovascular diseases persist as the main cause for morbidity and mortality worldwide.

The functional phenotype of each cellular unity is largely determined by its underlying gene expression leading to the recent increase in publications addressing the cardiac transcriptome. RNA is essential to biological processes in cells and cell-cell communication, providing critical information directly associated with cell phenotypes. Consequently, the transcriptome portrays a representation of the current biological pathways and processes in the examined material. Next generation sequencing (NGS) technologies have served as powerful tools to study genomic traits and provided key insights in various research or clinical fields (Wang et al., 2009; Garraway, 2013). To date, the most commonly used technique to decipher the transcriptional landscape is high-throughput RNA sequencing (RNA-Seq), which offers a quantitative and open system for profiling transcriptional expression at genome scale and hence provides a variety of applications. Several robust RNA-Seq protocols have since been developed, each with its distinctive purpose and (dis)advantages and rendering RNA-Seq almost mandatory for every molecular biology study.

The introduction of single-cell RNA-Seq (scRNA-Seq) has revolutionized genomic research and is yet another milestone to add to the list of accomplishments that include the completion of the Human Genome Project in 2003 (International Human Genome Sequencing Consortium, 2004). Furthermore, since its invention more than 10 years ago scRNA-Seq has made tremendous progress driven by the rapid development of innovative technologies and computational analysis methods, and more importantly the in-depth knowledge of biological processes. Its biggest advantage lies in its capability to look at the transcriptome of individual cells compared to conventional bulk techniques, which measure the average gene expression across cells in a sample. It is therefore not surprising that the cardiovascular field quickly started to integrate transcriptomic techniques into their research. The most recent studies already identified a large heterogeneity among cardiac cell types and during cellular differentiation, allowing for the discovery of novel genes involved in the complex connectivity network.

Despite all these innovations, it is essential to make an informed choice regarding the appropriate technique for the study of interest, especially considering myocardial tissue. However, many researchers in the field do not yet have a complete apprehension of the various technologies and their benefits and pitfalls. Therefore, here we provide a primer for researchers interested in transcriptomics using NGS technologies.

## Sequencing Methodologies

### Bulk RNA Sequencing

For over a decade, researchers all over the world have used the conventional bulk sequencing methods on RNA extracted from a population of cells to study gene expression changes in different tissues (Cloonan et al., 2008; Lister et al., 2008; Mortazavi et al., 2008; Nagalakshmi et al., 2008; Wilhelm et al., 2008). Since then, the system has been optimized for different types of RNAs and qualities of starting material. In general, bulk RNA-Seq refers to every sequencing approach that relies on averaged gene expression from a population of cells to reveal RNA presence as well as quantity in a sample of cells during the time of measurement. Therefore, bulk-based approaches can identify differences between sample conditions. Bulk RNA-Seq is not particularly limited by technical applications to the heart, nevertheless there are many criteria to consider when choosing bulk sequencing to ensure high-quality data.

#### Sample and Library Preparation

Several steps can make a difference on data quality during bulk RNA-Seq pipelines. For instance, sample and library preparation will have a direct effect on the outcome of the analysis. Their workflow can be further subdivided into RNA isolation, RNA depletion and cDNA synthesis. Unfortunately, their single stranded nature makes RNA very unstable, and susceptible to hydrolysis and heat degradation. To ensure the optimal conditions before sequencing RNA quality must be assessed, which is commonly done using the RNA Integrity Number (RIN) with a value between 1 (low quality) and 10 (high quality) (Schroeder et al., 2006). A RIN value over six is considered good enough for sequencing (Kukurba and Montgomery, 2015), confirming the availability of high-quality RNA. Unfortunately, samples obtained from human biopsies or paraffin embedded tissues can have an adverse effect on the quality of RNA retained. Of note, even frozen RNA will lose quality over the years (Seelenfreund et al., 2014) and therefore, the RIN should always be assessed right before library preparation. In general, bulk RNA-Seq requires a minimal amount of RNA as input, but certain methodologies require more.

“Bulk” refers to the total source of RNA in a cell population allowing in depth analysis and therefore all molecules of the transcriptome can be evaluated using bulk sequencing. Interestingly, total RNA can be sequenced, or specific types of RNA can be isolated beforehand from the total RNA pool, which is composed of ribosomal RNA (rRNA), pre-mRNA and the different classes of non-coding RNA (ncRNA). Various methodologies have been developed to selectively deplete or enrich a specific type of RNA molecule before or during library preparation (Accerbi et al., 2010). It is recommended to remove rRNA transcripts before library construction due to its over presentation in the cell. Otherwise, rRNAs will overwhelm most sequencing reads and leading to an overall reduction in sequencing depth and detection of less-abundant RNAs, such as many ncRNAs (Chu and Corey, 2012)*.* When focusing on protein-coding RNA molecules, many protocols aim to enrich for polyadenylated RNA by using poly(T) oligos targeting the poly(A)-tail of mRNA instead of depleting rRNA. In projects focusing on ncRNA, rRNA-depletion seems to be a more appropriate choice, which allows also quantification of pre-mRNA that has not been post-transcriptionally modified. Of note, slight differences exist between rRNA-depletion protocols in terms of rRNA removal efficiency and differential coverage of small genes, which should be investigated before selecting a method (Huang et al., 2011)*.*

As mentioned above, selective protocols have been developed to target specific RNA molecules, such as small RNA, which are key regulators of gene expression. As small RNAs are lowly abundant, short in length (15–30 nt) and lacks polyadenylation, a separate strategy is often preferred to isolate and profile these RNAs using commercially available extraction kits similar to total RNA isolation protocols. Most kits involve isolation of small RNAs by size fractionation using gel electrophoresis or capture using silica spin columns. After isolation of small or other preferred RNA species from the total RNA, the sample is ready for library generation, which is universal for most RNA-Seq preparations. This step contains by fragmentation, reverse transcription into double stranded cDNA and adapter ligation. Even though there might be small differences in library preparation depending on the used platform/platform provider, but overall, these steps are generally applied. The fragmentation of reads, or in simple terms cutting the total RNA into smaller pieces, can be achieved by physical (e.g., sonication), enzymatic (e.g., RNAse II, transposase) or chemical (e.g., heat) means. The subsequent cDNA synthesis is essential for stability and improves confidence of base calling, which decreases with read length. Adapter ligation is necessary for sequencing, but also determines the next step, single-end or paired-end (PE) sequencing. In brief, single-end reads will only be sequenced from on end, either 3′ or 5′. PE sequencing starts to sequence at one end, and after a predetermined read length (∼100 bp), it stops and starts to read from the opposite end of the fragment, thus generating two reads per transcript (Cheng et al., 2020). Hence PE keeps strand information and is therefore more suited for studies of isoforms (Piskol et al., 2013).

Short fragmented sequencing is the most commonly used method, but involves a higher false-discovery rate in terms of reconstruction and read counting. To overcome this, long-read technologies have been developed to enable sequencing of entire transcripts from 5 ´end to 3 ´end, thus providing improved coverage. Companies such as PacBio and Oxford Nanopore technologies have provided direct sequencing of RNA platforms that belong to the Third Generation of sequencing and are capable to generate long-reads of around 10 kb. These long reads allow coverage of entire transcripts and improve the identification of new splicing events and eliminate amplification bias. However, a downside to these technologies is a lower sensitivity due to the high number of discarded reads during the pre-processing step, due to the high RNA degradation rate, which ameliorates the integrity of the transcripts and jeopardizes the accuracy of the reads and data analysis. Furthermore, RNA sequencing lacks the proofreading exonuclease activity as well as strand directionality information leading to elevated error rates, which are around 8–10%, compared to <1% of Illumina sequencers (Kovaka et al., 2019) direct (Levin et al., 2010; Venkataraman et al., 2018).

Conclusively, there are various sequencing methodologies available that focus on sequencing specific RNA molecules or targeted regions. This is further attributed by specialized computational pipelines focusing on specific RNA classes. Therefore, prior knowledge of available and current sequencing technologies as well as study designing with utmost care, will greatly benefit the impact and quality of produced results.

### Single-Cell Sequencing

In 2013, Nature Methods titled scRNA-Seq as one of the most anticipated technologies of the year (Method of the Year 2013, 2014) with follow up nomination as technology of the year in 2019 due to its key role in the identification of cell types and functions, in addition to possible simultaneously multi-omics approaches in the future, highlighting its extensive role in genomic research.

Over the last couple of years, scRNA-Seq has had a massive effect on research. The reason is simple - while bulk RNA-Seq can measure the average gene expression across cells in a sample, identify differences between sample conditions and give a representation of highly regulated pathways, it fails to demonstrate the individual complexity of each cell and heterogeneity of tissues. Furthermore, some cell populations have high degrees of cellular and transcriptomic heterogeneity due to different cell types or indiscriminate states. The advent of scRNA-Seq technologies has addressed most of these limitations by facilitating the analysis of the transcriptome of every cell in each sample (Shapiro et al., 2013; Islam et al., 2014). This has enabled the creation of cell atlases (Regev et al., 2017; Cusanovich et al., 2018; Han et al., 2018; Tabula Muris Consortium., 2018; Cui et al., 2019; Litviňuková et al., 2020; Suryawanshi et al., 2020) at unprecedented resolution, the analysis of thousands of cells in parallel and even allows integration of chromatin status and multimodal analysis.

As a result, scRNA-Seq has shown to be a helpful technique for identifying cell subpopulations and elucidating dynamic cellular transitions during development and differentiation with an unparalleled level of detail and accuracy (Lafzi et al., 2018; Potter, 2018).

Several scRNA-Seq methods and technologies have been developed in recent years, all having different criteria regarding RNA transcript length, number of captured cells and read depth per cell (Svensson et al., 2018). Therefore, a specific scRNA-seq technology may be more beneficial for certain types of material than others, but all of them share similar workflows: samples preparation, single-cell capture, transcription and amplification, library preparation, sequencing and analysis (Potter, 2018). In general, there are three main technologies of scRNA-Seq: plate-, microfluidic- and droplet-based. In this section we will first explain the basic concepts of these currently used platforms and then their applications in cardiovascular science.

#### Single-Cell Dissociation and Capture

Sample preparation, similar to bulk RNA-Seq, is critical to enable single-cell capture and high-quality data. It starts with dissociation of material or tissue into a single cell form in order to extract cellular RNA. The main challenges included in this step are the fragility of the starting sample, physical stress, the choice of buffers, the duration of cell dissociation and the yield of individual and viable cells as most protocols require living cells (Lafzi et al., 2018; Nguyen et al., 2018). Cell isolation is a delicate process and obtaining a high-quality sample is of immense importance for a study to be successful. Therefore, single-cell dissociation is mostly achieved enzymatically using optimized protocols to limit cell lysis and loss of valuable RNAs. However, for all techniques, it is crucial to keep the processing time to a minimum to avoid cell damage and to prevent the unnecessary expression of stress-related genes, thereby altering original cellular transcriptomic profiles. It is noteworthy that cell isolation and single cell solution creation highly depends on the tissue in question. Primary samples, particularly when obtained from patients during an intervention, tend to be snap frozen, which is mostly incompatible with downstream analysis such as scRNA-Seq preparation from live cells. Creating single-cell solutions from snap frozen samples normally yield a lower overall number of cells and less viable cells (Reichard and Asosingh, 2019). Therefore, the most recommended for a single molecule approach is single nuclei sequencing (more information below) (Mimpen et al., 2021). Even if fresh material is available, certain tissue characteristics, e.g., as observed in disease depended fibrosis or scaring, can severely affect the digestion procedure. Therefore, we suggest thoughtful optimization to ensure high-quality scRNA-Seq data from primary tissue (Litvinukova et al., 2018; Vieira Braga and Miragaia, 2019).

After successful dissociation, single cells must be captured. As mentioned above, there are currently multiple techniques, and the choice largely depends on the sample of interest. However, capturing a high number of cells in the range of ≥10,000 cells will ensure significant improvements in data quality. One of the most critical properties determining the capture method is cell size. Plate-based methods incorporate either the use of micropipettes or fluorescence-activated cell sorting (FACS), guiding a single cell per well of a 96-well or 384-well plate. This method is mostly unaffected by size and has the option of long-term storage, as each cell is directly lysed within a well (Gladka et al., 2018; Hwang et al., 2018). However, since most steps have to be performed per well, the number of cells that can be processed at once is limited.

Microfluidic-based methods on the other hand separate the cells using narrow parallel microfluidic channels, where cell capture, cell lysis, reverse transcription, and multiplexing take place within an integrated fluidic circuit chip. In addition, a nice feature of this technique is the possibility to view capture cells before reverse transcription. An advantage of these methods is the high recovery/capture rate; however, they do require homogeneity in cell size. Furthermore, most microfluidic platforms require an input of >10.000 cells and are limited due to their restricted number of capture sites per microfluidic array.

Droplet-based approaches require the encapsulating of single cells into oil droplets with cell-specific barcoding. Due to its massive parallelization, ∼10.000 cells per sample can be captured within each run. For instance, 10X Genomics recommends a starting point of at least ∼1,600 cells per reaction for 3’ analyses, resulting in a recovery of ∼1.000 cells, and a multiplet rate of ∼0.8% (XGenomics, 2019). However, these platforms are limited to cell sizes less than 30 μm in diameter, and cells larger than that will clog the nozzle of the droplet system. Moreover, droplet- and microfluidic-based approaches have the need for living populations of single cells as input. Additionally, it is advised to remove cell aggregates, dead cells, and cell debris, before capturing to ensure a high percentage of viability among the selected population.

ICELL8 (Goldstein et al., 2017) is another promising platform, which is nanowell-based and therefore mostly similar to plate-based methods but with certain advantages. It utilizes a large-bore nozzle dispenser to distribute 1,000–1,800 single cells from diluted cell suspensions into 5.184 nanowells. Nanowell-specific barcoded are used to track sequencing data to its originator cell. Furthermore, ICELL8 has an imaging system visualizing all nanowells and by using fluorescence signals, it allows the differentiation of viable from dead cells and single-from multi-cells. A summary of main features of available scRNA-seq platforms can be found in Table 1.

**TABLE 1:** Main features of available single-cell methodologies.

Method	SmartSeq/C1	SmartSeq/C2	CEL-seq/C1	Drop-seq	MARS-seq	SCRB-seq	10X genomics	Wafergen/ICELL8
Cell Input	≥ 10,000	no limitation	≥ 10,000	≥ 10,000	≥ 10,000	no limitation	≥ 20,000	no limitation
UMI length (bp)	No	No	6	8	8	10	10	10
#Genes/Cell	**	***	**	*	*	**	***	***
Accuracy	**	****	*	***	*	**	**	**
cDNA coverage	Full Length	Full Length	3′ counting	3′ counting	3′ counting	3′ counting	3′ counting and 5′ counting	Full Length
Target Depth (per cell)	1.00E+06	1.00E+06	1.00E+04-1.00E+05	1.00E+04-1.00E+05	1.00E+04-1.00E+05	1.00E+04	1.00E+04-1.00E+05	1.00E+06
Amplification Type	PCR	PCR	IVT	PCR	IVT	PCR	PCR	PCR
Cost/Cell	****	***	***	**	***	***	*	***
Cell Size	homogenous (5–25 μm)	indepent	homogenous (5–25 μm)	homogenous (<25 μm)	indepent	indepent	homogenous (<30 μm)	homogenous (<5–100 µm)
Year	2012	2013	2014	2015	2014	2014	2017	2017
References	Ramsköld et al. (2012)	Picelli et al. (2014)	Grün et al. (2014)	Jaitin et al. (2014)	Macosko et al. (2015)	Soumillon et al. (2014)	Zheng et al. (2017)	Goldstein et al. (2017)

Abbreviations; scRNA-Seq single cell RNA, sequencing; Smart-Seq novel full-transcriptome mRNA-sequencing protocol; CEL-Seq cell expression by linear amplification and sequencing; Drop-Seq droplet sequencing; IVT, *in vitro* transcription; UMI, unique molecular modifier; MARS-Seq massive parallel RNA, single cell sequencing framework.

#### Sequencing of Single-Cells

Post cell capture, the individual cells/droplets are lysed, converted into cDNA *via* reverse transcription and finally sequenced. These steps depend highly on the capturing method chosen and show differences on multiple sequencing aspects. In terms of sequencing, plate-based methods rely on individual reverse transcription in each well, which can limit throughput and increase noise in downstream analyses. However, these methods allow full-length transcript sequencing, preferable for the identification of isoform splicing. Overall, plate-based platforms generally have high sensitivity and can reliably quantify up to 10,000 genes per cell.

Microfluidic-base methods are high in sensitivity and can use full-length transcript sequencing as well. Automatic systems like the Fluidigm C1 originally using Smart-Seq (Ramsköld et al., 2012; Picelli, 2019) before moving on the CEL-Seq method, were among the first scRNA-Seq platforms, increasing the transcript sensitivity and gene detection (Hashimshony et al., 2016). Smart-Seq methods can capture full-length transcripts (Ramsköld et al., 2012; Picelli et al., 2014) and therefore allowing the possibility to analysis alternate isoform splicing, which is further developed as a primary focus in Smart-Seq3 (Hagemann-Jensen et al., 2020). However, in comparison CEL-seq2 is limited to 3′-end reading and, therefore, cannot detect alternatively spliced isoforms, microRNAs or other non-polyadenylated transcripts.

Droplet-based methods have various advantages such as their massive parallelization, but they are size limited. Some commercial systems, such as the 10x Genomics Chromium, enable high-throughput processing by 3′- or 5′-end sequencing, but unfortunately also show reduced transcript recovery rates compared to other methods (Papalexi and Satija, 2018) with a read-depth of 10^4^ to 10^5^ per cell (Hwang et al., 2018). Even though this seems to be a drawback compared to the other techniques, droplet-based methods remain sufficient for the large-scale profiling of complex heterogeneous samples.

The actual sequencing of scRNA-Seq material can be performed on commonly used machines, and each single cell platform has particular features and come with individual recommendations to ensure best sequencing results and demultiplexing compatibilities (matching single cell tags with individual cells and their RNA content). However, one current limitation of high throughput scRNA-Seq, is that it generates either 3′ or 5′ sequence information via short length sequencing. This restricts analysis on splicing and sequence heterogeneity for most of the transcripts. As mentioned briefly above, short read sequencing involves fragmentation, so originally the transcripts are indeed full length and could theoretically undergo direct RNA sequencing using Third Generation Sequencing. i.e., Oxford Nanopore which is compatible with certain 10x Genomics protocols. Conversely, protocols for full or long length sequencing in single cell approaches are basically absent. This is largely due to technical and predominantly downstream analysis hardships, including the high error rate of these platforms. Nevertheless, there are methods, which attempt to correct errors and integrate single cell long read sequencing (Volden et al., 2018; Singh et al., 2019; Lebrigand et al., 2020; Zhang et al., 2021). For example, the rolling circle to concatemeric consensus (R2C2) method can produce full-length cDNA sequences, achieving ∼98% (Volden et al., 2018; Cole et al., 2020; Volden and Christopher, 2021) or Single-cell Nanopore sequencing with UMIs (ScNaUmi-seq) with ∼99.8% accuracy (Lebrigand et al., 2020), compared to former ∼50%. However, some of these methods require sufficient sequencing coverage to call consensus reads or high sequencing depth.

#### Single-Cell Application on Cardiac Tissue

At least 50% of the cells in an adult human heart are cardiomyocytes (CM), providing the contraction needed to keep our heart beating. The human adult CM are >100 μm in diameter along the major axis which can cause problems for several steps within the creation of single cell data. Options for overcoming the size limitation are the use of fetal mammalian CM, which are smaller in size. Another more recent approach includes the sequencing of human induced pluripotent stem cells (hiPSCs), which have been differentiated into CM. While the verdict on their comparability to adult CM due to their immaturity and heterogeneity (Shi et al., 2017; Paik et al., 2020) is still out, these cells have the advantage of smaller size and their non-invasive human origin.

In general, plate-based methods are not particularly limited by size, making them seem like a preferable platform for CMs. However, due to their expensive single-cell selection and limit in number of processed cells at a time, it has to be thoroughly evaluated. Furthermore, studies performed suggested the use of a FACS nozzle of 500 μm in diameter (Kannan et al., 2019) rather than the conventional 70–130 μm (Gladka et al., 2018) to ensure no terminal damage to the live CM, which may affect laser delays.

Even so, microfluidic- and plate-base systems are also commonly used for large and fragile cells, such as CMs. However, microfluidic-based systems would require a prior sorting via FACS into different cell types due to their vulnerability to heterogeneity and the difference in cell sizes within the heart. Furthermore, the elongated shape of CMs, in addition to unfortunate positioning could cause clogging of the system.

Unfortunately, droplet-based methods like the 10X Genomics Chromium, cannot cope with cells in the size range of mature adult CMs at present. However, as all these systems tend to evolve very quickly, continued protocol optimization and reductions in cost are to be expected soon.

### Single Nuclei Sequencing

Per definition single-nuclei RNA sequencing (snRNA-Seq) is not a technique by itself, but more a modification of the scRNA-Seq methodology. However, due to its unique contributions and applications to the cardiac field, the technique has earned an exclusive overview of its own.

Single-cell sequencing is a powerful tool allowing in-depth characterization of cell populations within complex tissues. However, as discussed in the previous sub-section, scRNA-Seq has two major limitations when applied to cardiovascular tissues: dissociation of material and cell size. ScRNA-Seq systems require dissociation of tissue material and especially gaining high-quality single-cell resolution of the adult mammalian heart is rather difficult. Secondly, technical limitations regarding capturing techniques, leading to an under representation of individual cell types (i.e., CM) due to their large cell size and irregular shape (Ackers-Johnson et al., 2018). There are few platforms for sequencing individual adult CMs and given that all of them are plate-based systems, the number of cells they can analyze is limited.

As the name suggests, snRNA-Seq utilizes only the nuclear component of single cells as input material, which is most appropriate when viable intact cells cannot be harvested from fresh tissue. Furthermore, there are other advantages of snRNA-Seq, when scRNA-Seq capturing methodologies cannot be applied. For instance, it allows the extraction of nuclei from frozen samples, which is extremely valuable for rare materials like the heart. Moreover, scRNA-Seq involves a dissociation step, which might damage sensitive cells while failing to release cells more tightly bound or those surrounded by a matrix. Although sampling a much larger number of cells can partially overcome this limitation, it may not always be financially feasible. Additionally, as mentioned before current enzymatic and mechanical methods for single-cell dissociation tend to introduce stress-induced transcriptional artefacts. Even with commercial nuclei extraction kits, the solution should be examined to ensure no big cells are present anymore. Afterwards, single-nuclei can be prepared to be captured with any of the above-described methodologies.

Nevertheless, there are some strategic limitations to be considered. While some studies have shown similar comparable transcriptomic results acquired from sc- and snRNA-Seq (Bakken et al., 2018), by utilizing RNA from within the nucleus only, a fair amount of the total RNA is excluded from the analysis, which could constitute a limiting factor in identifying dynamic cellular states.

Furthermore, taking single nuclei from frozen samples might result in false transcriptomic representation due to RNA degradation by freezing. Interestingly, CM can have a diploid or even polyploid as well as multinucleate nature (Landim-Vieira et al., 2020), which is not fully understood yet and therefore very valuable for future studies. In addition, extracting the nuclei removes the highly abundant mitochondria in CMs leading to possible alterations in gene expression or missing information when looking into network-based approaches.

### Integrational Approaches

While scRNA-Seq is currently the most widespread RNA-Seq technology, its prowess in deciphering the individual make-up of a single cell is just beginning to evolve. In recent years, many suppliers were able to integrate multi-omic approaches on the single cell level. Immunological studies have benefitted from paired B-cell or T-cell receptors, surface protein expression together with gene expression from a single cell (DeBerge et al., 2021). Single nuclei sequencing can be combined with ATAC (Assay for Transposase Accessible Chromatin) allowing for the analysis of chromatin accessibility at the single cell level, thus providing insights into cell types and states, and deeper understanding of gene regulatory mechanisms (Zhang et al., 2021). Further applications include proteomics (Cheung et al., 2021) and DNA methylation (Galvão and Kelsey, 2021). However, as most of these are still in their early stages including protocol optimization and integrational data analysis tools, it needs to be individually assessed whether specific research questions can be answered using these approaches (Jansen et al., 2019; Argelaguet et al., 2020). On the other side, computational approaches tried to leverage cell-type specific gene expression profiles from multiple scRNA-seq reference datasets to bulk RNA-Seq with the intention to apply cell compositions even to bulk RNA-Seq data. To date, multiple tools have been suggested, such as DESCEND (Wang et al., 2018), SCDC (Dong et al., 2021) and some others, but to our knowledge not one has been yet considered to be “state-of-art”.

### Spatial Transcriptomics

Identifying gene expression profiles at single-cell resolution has revolutionized the field of transcriptomics. Using single-cell or single-nuclei based approaches has made it possible to look at individual cells within complex tissues, gaining valuable biological insights into rare cellular properties, cell-to-cell variability, and tissue identity. Despite such advantages, these approaches are limited by technical criteria to a certain degree. In this review, it has already been demonstrated how dissociating tissue can be a difficulty. In addition, this process causes the loss of information regarding the spatial organization of cells and cell populations throughout a tissue causing limited or incomplete interpretation. In *in vivo* situations though, spatial-specific expression has an enormous impact on biological networks. Gaining knowledge of spatial information in addition to timing, and level of developmental gene expression allows the description of interactive biological networks, where each element is influenced by its surrounding microenvironment (Zheng et al., 2017). Therefore, the persevation of spatial information holds promising approaches of combining genomic, transcriptomic, and proteomic features while retaining positional information.

Various scRNA-Seq methodologies have been discussed in the section above, but spatial approaches also have made extraordinary progress in the recent years (Ståhl et al., 2016; Qian et al., 2020) and gained increasing popularity. In general, spatial techniques vary from *in situ* hybridization (e.g., smFISH, seqFISH) over *in situ* sequencing (e.g., BaristaSeq, STARmap) to *in situ* capturing technologies (e.g., ST, Slide-Seq). All of them have their designated place in research, *in situ* spatial methodologies hold the prospect of possible combination with scRNA-Seq technologies. While some of these technologies and their drawbacks and advantages have been reviewed before (Asp et al., 2020), this review will highlight a few of the spatial methodologies and their applicability to cardiovascular research.

#### *In situ* Capturing Technologies

The idea behind *in situ* capturing technologies is to capture RNA *in situ* and then perform sequencing *ex situ*, avoiding typical limitations of direct visualization (e.g., limited marker amount, fluorescence exposure) as well as allowing an unbiased analysis of the complete transcriptome. The first of such technology to be developed was the Spatial Transcriptomics (ST), published 2016 (Ståhl et al., 2016), which was the basis for all following techniques. The basic idea behind this technology is transferring thin tissue section onto glass slides coated with positional molecular barcodes and synthesized cDNA and therefore able to capture mRNA while still maintaining positional representation. In detail, these slides are coded with the barcoded RT primers which the tissue is fixed, stained, imaged and permeabilized upon. The mRNA molecules diffuse down to the slide surface during the permeabilization phase and hybridize locally to the RT primers. Afterwards, the tissue is removed, the RNA is captured and retrotranscribed *in situ* and the cDNA-mRNA complexes are used for library preparation in the following *ex situ* NGS. By overlaying the different images, the barcoded reads are superimposed back onto the tissue image*.*

This approach provided spatial information on the transcriptome of the tissue, but *the barcoded* regions were only 10 μm in diameter. Depending on the tissue or cells of interest, this could be enough to reach a single-cell resolution. To tackle this, 10X Genomics acquired ST and started to further develop it into “10X Visium” by the end of 2018 (Tirado-Lee, 2020). 10X achieved further improvements in terms of resolution (55 μm in diameter and smaller distance between barcoded regions) and protocol running time. In addition, 10X has further expanded by adding *Visium Gateway slides* to their Visium repertoire (Tirado-Lee, 2020). Interestingly, 10X Genomics announced a new update “Visium HD”, which supposedly offers visibility 400 times higher than the current Visium solution with improvements addressing RNA degradation problems during formalin fixed paraffin embeddings.

Another method using a similar approach, Slide-Seq, was introduced in 2019 (Rodriques et al., 2019), promising a higher spatial-resolution technique by using smaller barcoded beads (Vickovic et al., 2019)*.* Recently, Stickels et al. (2020b) published Slide-SeqV2, combining improvements in library generation, bead synthesis and array indexing resulting ∼10-fold greater RNA capture rate than its predecessor.

In March 2019, NanoString announced the commercial launch of their GeoMx Digital Spatial Profiling instrument (NanoString). In contrast, to previously mentioned platforms, GeoMx is capable of the highly multiplexed detection of mRNA targets in FFPE tissues (Merritt et al., 2020). Using multiple sections of a sample will help to acquire not only the spatial transcriptomic profiles but in addition also the spatial protein profiles. GeoMx can utilize regions in the range of 10–600 μm in diameter, which have to be selected manually. After selecting the regions of interest, fluorescence labeled antibodies used as morphology markers are then excited with UV light, triggering the release of either RNA target probes or antibodies for protein targeting, coupled with barcoded tags. Conveniently, using the NanoString’s nCounter instrument will assist to collect and quantify the tags from the regions of interest, resulting in a digital quantification of RNA expression with spatial context (Merritt et al., 2020). The workflow of selecting regions of interest makes it possible to analyze almost whole tissue sections*,* but the selection is still manual and therefore a biased process. However, despite increased advances leading to capture sensitivity and efficiency (Stickels et al., 2020a), scRNA-Seq data is still required to help mapping of cell types.

### Data Analysis and Computational Approaches

**FIGURE 1:**
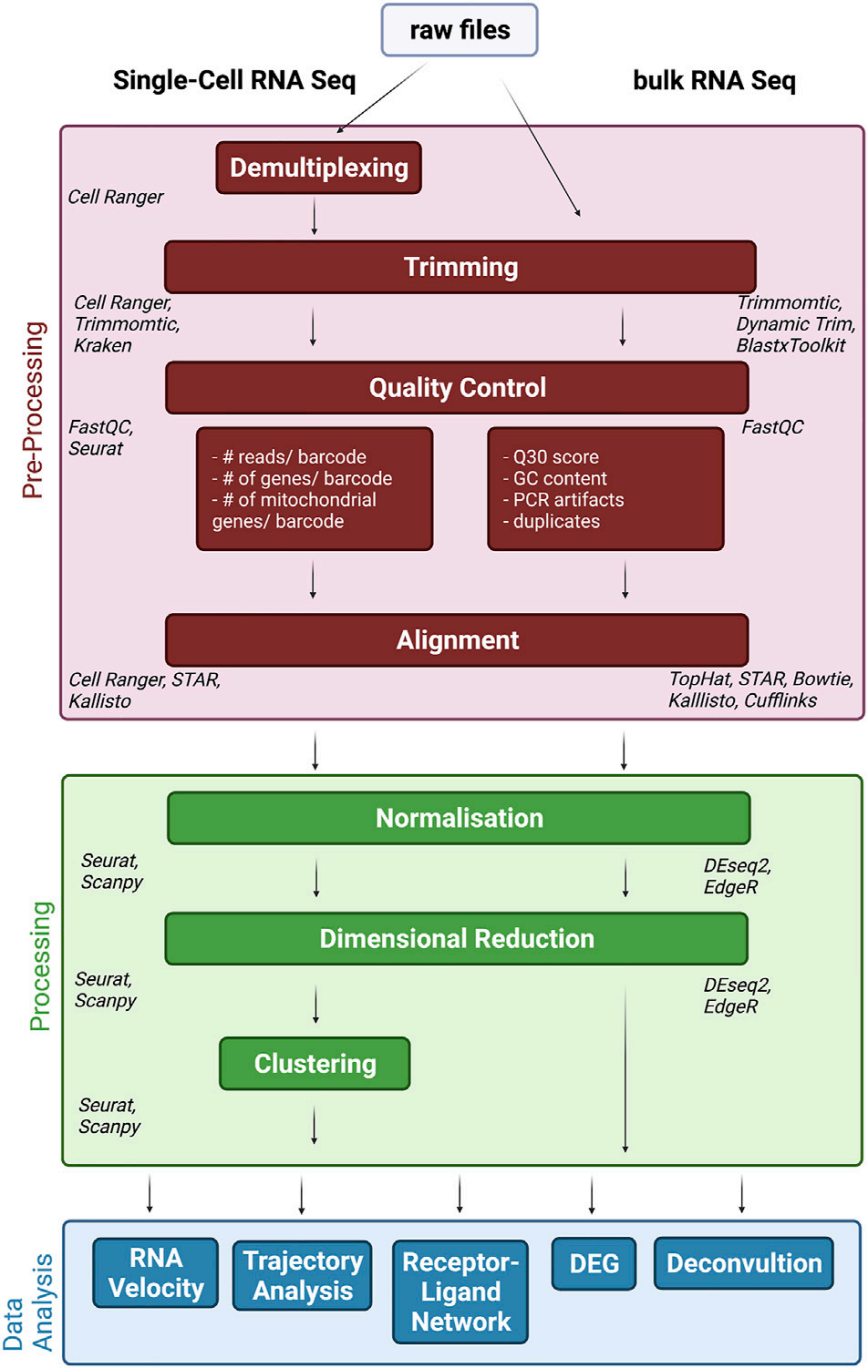
Data Processing and Analysis pipeline. After sequencing files need to be pre-, processed and analysis (individually colored) in order to interpret the data and put it into biological context. These steps are very similar for single-cell (left) and bulk (right) RNA-Seq approaches. Italics represent most common used tools for the specific tasks.

All above mentioned procedures have a tremendous effect on data quality. However, in order to draw meaningful (biological) conclusions, downstream data procession and analysis is necessary. In fact, data analysis should be taken into consideration when designing a study in the first place, to allow for a qualitative interpretation of the data. Therefore, here we discuss the basic steps of data processing, analysis and opportunities for downstream analysis (Figure 1). Of note, this section will not be separated into bulk and single-cell sequencing as both follow similar pipelines. In addition, many single-cell advances are actually adapted from existing bulk approaches. At this stage, we would like to refer to other publications addressing data analysis steps and challenges (Koch et al., 2018; Dal Molin and Di Camillo, 2019; Lähnemann et al., 2020) in single cell vs. bulk RNA approaches.

After sequencing the libraries, the raw files need to be pre-processed. This step includes demultiplexing, quality control, trimming and alignment/*de novo* assembly. Even though existing in both bulk and single cell analysis, demultiplexing refers to slightly different processes. In bulk RNA-Seq, a sequencing lane typically will contain a pool of barcoded samples, which therefore require demultiplexing. In scRNA-Seq, each cell is considered a sample and, in most protocols, defined by a cell-specific barcode tag to each read. Therefore, each library normally contains multiple cell barcodes, which need to be distinguished as a sample. This process is called demultiplexing and gives each cell in each sample its specific RNA content. From here on, the steps of processing are in common between approaches, which are meant to cut off primer/adapters or poor-quality nucleotides, alignment of reads to a reference genome and counting reads per region/gene, creating a gene count matrix. Due to the overall high input amount for bulk sequencing, a high coverage can be achieved, but unfortunately scRNA-Seq normally deals with to the low concentration of input RNA and results in a high number of zero reads counts. However, these “false” zero counts, such as dropout events or sparsity, may mask the biological zero counts due to the non-expression of a group of genes in specific cells (Eraslan et al., 2019). Moreover, this high number of zero read counts in the gene expression matrix ultimately results in the reconstruction of an incomplete transcriptome, which renders the study of the different cellular phenotypes very challenging (Wang J. et al., 2019). Sparsity can impede downstream analysis and remains a challenge. To overcome this loss of information, computational imputation or denoising was developed, i.e., MAGIC and scIMPUTE (Lafzi et al., 2018; van Dijk et al., 2018). The last step is normalization. There are different approaches to normalization depending on the software used, but most of them are equally accepted and their individual usage mostly depends on the intented downstream analysis. However, the methods developed for bulk RNA-Seq such as RPKM, FPKM and TMM were not able to successfully remove the effects of different sampling from scRNA-Seq datasets, due to the considerable technical noise present in such data. Therefore, new normalization methods, which are able to handle noisy data and take advantage of the presence of UMIs were developed (Townes and Irizarry, 2020).

Afterwards, the data can be processed in various ways. Dimensional reduction is one of the most commonly used features and helps to visualize data and to identify relevant genes. For bulk-based studies, principal component analysis (PCA) is the most common used feature. Although this also applies to single cell approaches, because of the high dimensionality of the data other methodologies, such as t-SNE and UMAP, are superior to PCA. Other limitations include errors introduced by the inevitable technical variability during sample processing steps, sequencing depth or pipetting accuracy, also known as batch effect.

These are not unique to one methodology and can be found in the datasets generated by both, bulk and scRNA-Seq. However, these confounding factors are more amplified in scRNA-Seq, which may lead to a mix of technical variations with biological variables. In bulk RNA-Seq, instead, these batch effects are smaller and do not result as systematic errors, as bigger sample sizes usually lead to large-scale sample preparation and sequencing in parallel. Therefore, batch effect correction in scRNA-Seq requires other features capable of handling these variations, for example nearest neighbor integration. Further typical analyses include clustering and differential expression gene (DEG) detection. Unsupervised clustering further helps to identify groups and subgroups within single cell datasets and to pinpoint relevant cell groups for further analysis. DEG analysis has been established as a powerful tool to discover different regulation between groups, which can reveal regulatory effects. Although DEG can be also used to make between-sample comparisons, it is usually applied to identify transcriptome signatures that differentiate between cell groups. However, cell group assignment is still constrained by ambiguities, which could mask biological differences. Therefore, an improved reference set for cell type assignment is necessary to make DEG analysis in single-cells more reliable.

However, scRNA-Seq also provided new bioinformatics tools, with RNA velocity and trajectory analysis being the most prominent features. Both aim to describe the dynamic changes among captured cell signatures. RNA Velocity takes advantage of the ratio between spliced and unspliced RNA to develop dynamical patterns with possible cell fate prediction, while the trajectory attempts to predict a path that certain cells undergo during specific conditions (i.e., differentiation). Especially, deconvolution of RNA seq data has gained increased recognition in the last year (Baron et al., 2016; Wang X. et al., 2019; Newman et al., 2019). Deconvolution attempts to use single cell datasets to deconvolute bulk datasets by leveraging cell-type specific gene expression profiles from multiple scRNA-seq reference datasets, and thus synergistically combines specific information gained from both, bulk and single cell datasets.

## Sequencing the Heart

### Bulk Sequencing in Cardiovascular Research

While extracting RNA from heart tissue has no particular limiting factor, human heart samples are highly valuable due to the many ethical restrictions, making them hard to obtain. Many cardiac studies are therefore performed using other mammalian models such as mice or human tissue-derived cell lines. Nevertheless, patients with heart disease can undergo surgery, allowing for ascertainment of cardiac biopsies, but the amount of tissue retrieved can be insufficient or compromised. However, healthy individuals normally do not usually tend to undergo biopsy, rendering the collection of healthy living heart tissue as controls close to impossible.

Nevertheless, RNA-based studies have provided important insights to cardiac research, especially helping to decipher ncRNA and their potential role as regulators of numerous cellular processes in the progression of cardiovascular diseases Transcriptomic studies using NGS enabled clear distinguishment between various cardiac cell types, such as CMs, endothelial cells, fibroblasts, and immune cells. Arguably one of the first studies that compared accuracy and speed of NGS with microarray analyses demonstrated that in the Gαq transgenic mouse cardiomyopathy model RNA sequencing was accurate and sensitive enough to detect abundant and even rare cardiac transcripts (Matkovich et al., 2010). Subsequent studies highlighted that even atrial and ventricular cells show clear significant differences in their transcriptomic profiles (Sehnert et al., 2002; Ng et al., 2010). While atrial CM are mostly described with *Myh4*/ALC1 and *Myh6*/αMHC, the ventricle expresses more *Myh3*/ELC, *Myh7*/βMHC and *Myl2* myosin genes (Ng et al., 2010). In depth reports of the current knowledge on the different molecules in cardiac cell subtypes have been reviewed before (Zhou et al., 2018; Colpaert and Calore, 2019; Siasos et al., 2020)*.* NGS was essential to reveal that >80% of the genome is transcribed in various classes of RNA with protein-coding transcripts only accounting for max. 3% of the genome, and the remainder of transcripts lacking coding ability (Dunham et al., 2012). Many of these noncoding transcripts are functionally active RNA molecules that can be subdivided into small noncoding RNAs (<200 nt), such as microRNAs (miRs), and longer noncoding RNAs (>200 nt) that include ribosomal RNAs, natural antisense transcripts and long noncoding RNAs (lncRNAs) (Engreitz et al., 2016). NGS has helped to discover cardiac microRNAs (Matkovich et al., 2010; Leptidis et al., 2013) and lncRNAs (Matkovich et al., 2014; Yang et al., 2014) in the developing and diseased human heart and how they can potentially function as therapeutic target molecules. While these studies contributed significantly to our current understanding of cardiac (patho)physiology, they failed to address the importance of cardiac cellular heterogeneity and cell-cell interactions.

### Single-Molecule Sequencing and Cardiovascular Research

ScRNA-Seq is able to look at cell compositions in an unbiased fashion. In 2016, two labs first reported the use of scRNA-Seq on embryonic mouse hearts by investigating lineage-specific gene programs underling early cardiac development (DeLaughter et al., 2016; Li et al., 2016). To date, multiple researchers have used scRNA-based techniques on various cardiac materials allowing to look at cell populations at a single-cell resolution, and started a new age of transcriptomics including the generation of transcriptomic and epigenetic cell atlases of adult mice (Cusanovich et al., 2018; Han et al., 2018; Tabula Muris Consortium, 2018) and humans (Regev et al., 2017; Cui et al., 2019; Litviňuková et al., 2020; Suryawanshi et al., 2020). Investigations deciphering the heterogeneity and subpopulations of cell types define region- or disease-specific gene expression profiles not only benefit cell atlases but also help to identify novel molecular mechanisms relevant for cardiac disease and new therapies. For instance, Li et al. (2019) investigated cardiac endothelial cells following ischemic injury and were able to show clonal proliferation of resident endothelial cells post-myocardial infarction.

Single-cell approaches, however, do not stop here. The heart is a multi-cellular organ and the interaction between cell of ligands and receptors on target gene expression is not yet fully understood. Browaeys et al. recently developed a tool for modelling intercellular communication on the single-cell level by linking ligands to target genes. This as well as other promising tools enable investigation of possible signaling mediators in addition to gene interaction by expression profiling (Browaeys et al., 2020; Cang and Nie, 2020; Efremova et al., 2020; Gladka, 2020; Jin et al., 2021). Furthermore, network and trajectory analyses on single-cell data led to the identification of fibroblasts as a critical constituent in promoting cardiomyocyte maturity (See et al., 2017) and regulatory interactions between transcription factors and target genes (Jackson et al., 2020). Another recent study by Litviňuková et al. (2020) provided comprehensive transcriptomic data on six distinct cardiac regions of the adult human heart using both sc- and snRNA-Seq technologies. Similar to other reports, they observed the cellular heterogeneity of cardiomyocytes, pericytes, and fibroblasts, and additionally, they analyzed cell-to-cell interactions suggesting a direct interaction through NOTCH2. Another study using microfluidics approaches in healthy human donors was able to identify and study the cellular and transcriptional diversity in a healthy heart (Tucker et al., 2020). The ICELL8 platforms claims to be able to deal with a wide range of sizes, while still making high throughput sequencing possible. Wang *et al.* used this platform on 21,422 single cells-including CMs and non-CMs from normal, failed and partially recovered adult human hearts to reveal inter- and intra-compartmental heterogeneity in response to stress as well as CM contractility and metabolism as the source of changes in heart function (Wang et al., 2020).

Taken together, an increasing number of studies have used methodologies on single cell level to shed light on the roles of various cell populations within in the mammalian heart helping future cardiac research and advances with their findings.

### Spatial Transcriptomics in Cardiovascular Research

Interpreting single-cell properties can be difficult (Asp et al., 2020; Stickels et al., 2020a), but by adding spatial context to the transcriptome it will greatly benefit the current understanding of many biological networks. However, the main hurdle for these spatial methods is restricted RNA capture efficiency, which becomes increasingly more challenging with higher resolution. Even though spatial transcriptomics has proved to be a valuable source of transcriptomic information, spatial transcriptomics in the heart, cardiac development, and regional changes in gene expression during heart maturation are strong areas of great interest for the future.

Two studies utilized spatial transcription offered by 10X Genomics and subsequently combined spatial transcriptomic data with scRNA-Seq through computational deconvolution methods in order to reconstitute detailed spatial transcriptomic maps of human myocardial infarction (Kuppe et al., 2020) and the development of the human heart (Asp et al., 2019). In 2017 the group of Asp et al. (2017) already used the ST method to study fetal markers and their localization within adult human cardiac biopsies and later on created a spatiotemporal atlas of the developing human heart in the first trimester (Asp et al., 2019) by combining scRNA-seq data with spatial transcriptomics and *in situ* sequencing. In 2020, Asp et al. (2020) used their approach of combing scRNA-Seq along with ST and *in situ* sequencing to uncover novel cell types, such as clusters of fibrosis-associated fibroblast-like cells and a subpopulation of cardiac muscle cells. Furthermore, they also visualized their result by integrating the spatial information into 3D transcriptional maps. A major caveat of this combined approach is that it maximizes the efficiency of using data information by providing both cell-type and regional information from tissues, e.g., human embryo tissue, that are not easily obtained.

## Conclusion

Over the last decades, studying the transcriptome became standard when investigating biological processes of physiological and pathological mechanisms. The increased interest in studying the RNA and its properties led to development of more advanced technology, which started from whole tissue, to cell population and finally reached single-cell level.

**FIGURE 2:**
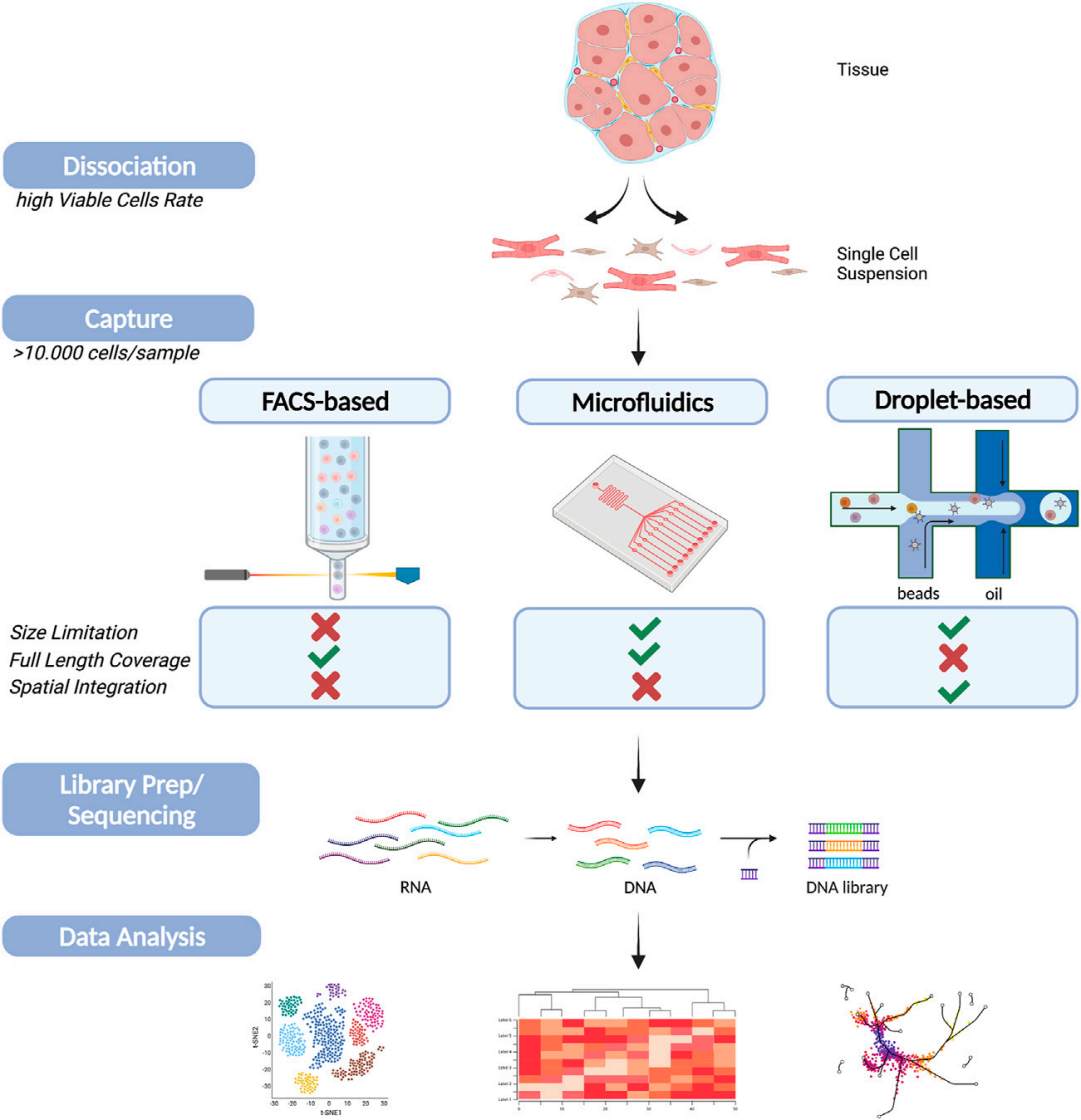
Schematic figure of single cell sequencing workflow on cardiac tissue. A summary of the main steps during scRNA-Seq experiments (Dissociation, Capture, Sequencing and Data Analysis). The main cell capturing techniques, FACS-based (left), Microfluidic (middle) and droplet-based (right), are schematically displayed including a comparison of their main advantages and disadvantages. Created with BioRender.com.

Sequencing techniques evolved quickly and increased our understanding of molecular signaling, intercellular communication networks and rare cell sub-populations. However, there is no one-size fits all approach; each technique has method-specific advantages and limitations. Hence researchers need to design their study with utmost care and choose a technique that aligns with their desired goals and sample availability. This furthermore includes the evaluation of multiple factors such as sample availability, sample preparation, platform advantages and disadvantages and sequencing attributes. This especially holds true for applications in cardiovascular research (Figure 2). Regardless, single-cell analysis has revolutionized our understanding of cardiovascular development and disease. The applications and insights that single-cell analysis enable in the investigation of cell subpopulation variants and differentiation in cardiovascular disease and heart development are invaluable to both basic and medical research. Furthermore, numerous cardiac single-cell atlases have been developed in multiple organisms and in multiple contexts including genetic variation, biological sex, and cardiac injury, all of which will provide useful resources for future work. Considering the deluge of recent advances in single cell analysis, it is becoming essential to develop and optimize methods for the integration of data of different types and from different sources. For instance, the development of antibody-based cell hashing, enabling multiplexing by indexing samples, holds future prospects, by lowering costs and allowing more complex experimental designs. In addition, multi-omic approaches trying to combine epigenetics, transcriptomic and proteomic aspects on single-cell level hold promising future innovations. Ultimately, scRNA-Seq technologies are expected to continue to develop rapidly with continuous improvement of experimental and analytical methods.

Taken together, single-cell analysis of the heart has revealed previously underappreciated cellular heterogeneity and the importance of intercellular communication. This diversity of cardiac cell types (and cell subtypes) acting together likely contributes to the homeostatic maintenance of cardiac tissue and is integral in the complex biological processes driving cell differentiation, cardiovascular development, disease, and regeneration. Finally, the use of single-cell level analytics will enable the definition of a healthy cardiac cell system and thereby better equip therapeutic pursuit toward the maintenance of this healthy cell system during physiological stress.
